# Effectiveness of a Chinese herbal medicine preparation in the treatment of cough in uncomplicated upper respiratory tract infection: a randomised double-blinded placebo-control trial

**DOI:** 10.1186/1745-9974-2-5

**Published:** 2006-06-22

**Authors:** Wong WCW, Lee A, Lam AT, Li KT, Leung CYM, Leung PC, Wong ELY, Tang JL

**Affiliations:** 1 Department of Community and Family Medicine, the Chinese University of Hong Kong, Hong Kong SAR, China; 2 Family Medicine Training Centre, Prince of Wales Hospital, New Territory East, Hong Kong SAR, China; 3 Clinical Trial Centre, Institute of Chinese Medicine, The Chinese University of Hong Kong, Hong Kong SAR, China

## Abstract

**Background:**

Rigorous scientific and well-designed clinical trials to evaluate the effect of traditional Chinese medicine (TCM) is lacking. We, therefore, designed this study to evaluate the effectiveness of a commonly used TCM preparation in treating acute cough of uncomplicated URTI in adults and to search for a safe, effective and affordable alternative treatment for this common condition.

**Methods:**

A randomised, double-blinded, placebo-control study comparing this TCM preparation with a placebo was conducted in 82 patients who attended the Family Medicine Training Centre, Prince of Wales Hospital, Hong Kong between November and December, 2003. The TCM herbal preparation includes nine commonly used TCM herbs for cough such as *chuanbei, fangfeng, jiegeng, gancao and baibu *(see Table 1) The treatment lasted for 5 days and patients were followed-up for another 6 days. Patients were asked to fill in a cough score and validated Leicester cough questionnaire (LCQ).

**Results:**

62 patients (75.6%) had completed the trial and no adverse effects were reported. Both intervened and control groups had improved in cough score and LCQ in the follow-up period, despite no overall statistical significance was observed in the differences of scores between the two groups. Women taking TCM had significantly fewer problems with sputum production (p = 0.03) and older subjects (>35 years of age) reported a significant improvement in hoarseness (p = 0.05) when compared to those using placebo.

**Conclusion:**

TCM was well-tolerated and received among the Hong Kong Chinese population. This TCM preparation appeared to have some benefits in the treatment of cough. Future research on TCM should concentrate more on commonly encountered conditions such as UTRI and cough. Our experience on the sensitivity of assessment tools used in detecting subtle differences in an otherwise self-limiting illness and clinical trial methodology when applying the underlying theory of how TCM works in disease management was invaluable.

**Table 1:** The components of TCM formulary in treating acute cough of uncomplicated URTIs

Bulbus Fritillariae Cirrhosae	27.3%
Herba Schizonepetae	10.5%
Radix Ledebouriellar	10.5%
Radix Platycodi	10.5%
Radix Glycyrrhizae	4.4%
Radix Asteris	10.5%
Radix Stemonae	10.5%
Rhizoma Cynanchi Stannotonii	10.5%
Pericarpium Citri Reticulatae	5.3%

## Background

Acute cough is a common presentation of upper respiratory tract infections (URTI) encountered in general practice [1]. In Australia in 1999, cough was treated in 7.5% of general consultation [2]. Cough can lead to high morbidity and cause debilitating symptoms such as exhaustion, insomnia, hoarseness, musculoskeletal pain, sweating and even urinary incontinence (3,4). The pressure produced during coughing could also potentially cause some kind of complication in nearly all organ systems [3]. More importantly, cough can be so profound that it may have an adverse effect on the patient's quality of life [4].

In 1994, over-the-counter sales of anti-tussives products in the United States was worth US$19 billions, which accounted for 38–50% of all respiratory sales [5]. The retail sale of cough mixtures in the United Kingdom rose by an annul rate of 3% to ₤94 m in 1999 [6]. Statistics from Pharmacy of the Department of Health in Hong Kong showed that their outpatients alone had consumed 370,000 liters of anti-tussives worth over 2 million Hong Kong dollars (US$1 = HK$7.8) in 2000 [7].

However, the effectiveness of anti-tussive in western medicine remains doubtful despite its large market and wide consumption. Only a small number of clinical trials investigating the anti-tussives so that evidence on their effectiveness is rather limited. Schroeder et al. [8] published a systematic review of all randomised controlled trials on various types of anti-tussives in 2002. They identified five trials tested for anti-tussives with placebo. Two on codeine and found none was more effective than placebo. One of two studies of dextromethorphan [9] favoured active treatment over placebo whereas the other found no significant effect. Moguisteine (one trial) led to mean differences in cough scores of about 0.5 in groups with severe cough on days 2 and 3 (P < 0.05), but there were no differences between groups at final follow up [10].

It is well known that not every ill person consults a health care professional. [11] Social and cultural factors may influence the pattern of symptomatology and phenomenology [12]. Patients disappointed with ineffective conventional treatments and naturally look for alternatives. Traditional Chinese Medicine (TCM) has been practiced in China for over 2000 years: Chinese patients take TCM for chronic health problems and they may also do that for some acute self-limiting problems [11]. TCM is considered to be a very acceptable alternative in Hong Kong and a sizable segment of the population consults TCM practitioners for their health problems [13]. In one survey, nearly half had previously consulted a TCM practitioner [14]. This is partly a cultural phenomenon but dissatisfaction with other forms of health care as in the case of cough was a commonly cited reason for resorting to TCM treatment [15].

We therefore designed this study to evaluate the effectiveness of a TCM formulary in treating acute cough of uncomplicated URTIs in adults. TCM used in this study was extracted from nine commonly used herbs in treating cough and, their functions and side effects were well documented [16]. Literature search was performed and the formulary was recommended by from a panel of three experienced Chinese herbalists. The nine ingredients used in this formulary are shown in Table 1. Bulbus Fritillariae Cirrhossae is the commonly used herb for the treatment of cough and it has been used for many centuries [17]. Animal studies showed that some alkaloids (imperialine, verticine and verticinone) extracted from Bulbus Fritilariae Cirrhossae acts like muscarinic receptor antagonist and are more potent than salbutamol and diphenhydramine in relaxing isolated rat trachea and bronchi [18]. Another major ingredient Radix platycodi has both anti-tussive and expectorant activities including the promotion of salivary and bronchial secretions [17]. Pericarpium Citri Reticulatae has expectorant activities and broncho-dilatative effect [17].

## Methods

### Study design

This was a single-centre, randomised, double blind, placebo-controlled and parallel study comparing TCM with placebo in patients who had presented with cough resulting from uncomplicated upper respiratory tract infections.

### Study patients

Patients were eligible for the study if they were over 18 years old, had cough due to clinically diagnosed URTIs that did not require antibiotics, not allergic to fexofenadine (Telfast^®^), not on other concurrent alternative medications for cough and were mentally capable to give an informed written consent and willing to comply with study requirements. We excluded patients who were pregnant or breastfeeding, current smokers, had lung disease (include asthma or chronic obstructive arirway disease) or cardiac disease (including valvular heart disease), had concurrent gastrointestinal symptoms such as nausea, vomiting, abdominal pain or diarrhoea) or if they were illiterate and had difficulties in filling in the diary.

### Study organisation

Patients were recruited from 17 Nov 2003 to 23 Dec 2003 at Prince of Wales Hospital Staff Clinic which mainly served Hospital Authority (HA) staff as well as their dependents such as spouse and children in New Territory East, one of the 6 districts in Hong Kong. Staff and students from the medical faculty might also attend. It mainly provided general medical consultations, specialty referrals, chronic disease management and pre-employment health checks.

### Study medication and dosage

TCM used in this study and the matching placebo were manufactured by the Hong Kong Institute of Biotechnology Ltd, based on Good Manufacturing Practice. The TCM powder using extract granules had been formulated into uniform tablets under the supervision of the Institute of Chinese Medicine at the Chinese University of Hong Kong. The dosage of study drug was 3 tablets (500 mg per tablet) three times a day.

### Randomisation

Randomisation and allocation was taken place on patients' first visit at the Staff Clinic. Informed consent was obtained according to the local laws and the Good Clinical Practices Guidelines, prior to the enrolment in this study and assignment of the subject study number. Subjects were given information regarding the nature, significance and scope of the study, tests to be performed and potential risks. They were also informed about their right to revoke their consent at any time without obligation to explain the reason and without prejudice to their further treatment.

### Outcome measures and data analysis

Treatment period lasted for 5 days. During which, clinical assessments including history, examination and tests (if necessary) were performed at day 4 and day 7. The participants were asked to fill a questionnaire to grade the severity of a range of symptoms related to cough and the functional disturbance of cough is measured by LCQ, which had been validated and permission to use it in this study from the original author was obtained. The first primary safety outcome is tolerability, which was defined as a permanent discontinuation of the mixture of TCM as the result of an adverse event. The second efficacy outcomes were a change in the cough symptom score and in the vitality status. Subjects were encouraged to withdraw from the trial and to be treated accordingly if there were any signs of deterioration in clinical presentation. This study was done on intention-to-treat basis that patients initially treated but subsequently dropouts were included in the final analysis.

Group data were expressed as the frequency unless otherwise specified. To analyse differences in the baseline parameters between TCM and placebo groups, student t-test was performed. The statistical significance of change differences between two study groups was tested by the Mann-Whitney U test in the comparison of cough symptoms and by the student t-test in the comparison of the results of quality of life scores. Subgroup analysis of age (those older than 35) and sex were performed using Mann-Whitney U test. All statistical tests were 2 sided and exact values for the rank sum. Data entry and analyses were performed with the SPSS software package.

## Results

Of the total numbers of 141 subjects screened, 81 consented to participate the study. Reasons for refusal included: Not willing to take TCM (41.7%), not available for study (13.3%), not willing to receive placebo (10%), not willing to take tablet (1.7%), not willing to do the questionnaire (1.7%), not interested in the study (1.7%), western medicine to TCM preferred (1.7%) and antitussive requested (1.7%). The baseline characteristics of patients in the intervened and control groups are shown in table 2.

**Table 2:** Characteristics of participants and dropouts in two study groups

	**Participants**		**Dropouts**	
	
	TCM group N (%)	Placebo group N (%)	TCM group N (%)	Placebo group N (%)
**Characteristic **	N = 41	N = 40	N = 11	N = 8

Sex				
Male	8 (19.5)	5(12.5)	3 (27)	2 (25)
Female	33 (80.5)	35 (87.5)	8 (73)	6 (75)

Marital status				
Single	16 (39.0)	12 (30.0)	2 (18)	4 (50)
Married	25 (61.0)	28 (70.0)	9 (82)	4 (50)

Education level				
< Primary level	5 (12.2)	3 (7.5)	1 (9)	0
< F.5	5 (12.2)	3 (7.5)	6(55)	5(63)
> F.5	16 (39.0)	17 (42.5)	1(9)	0
University level or above	15 (36.6)	17 (42.5)	3(27)	3(37)

Status				
HA/CU staff	36 (87.8)	38 (95.0)	11 (100)	8 (100)
Dependant of HA/CU staff	5 (12.2)	1 (2.5)	0	0

Occupation				
HA staff:	36 (44.4)	39 (48.1)		
Nurses	8 (19.5)	11 (27.5)	2(18)	1 (12)
Allied health	5 (12.2)	4 (10)	1(9)	3(38)
Technician/Researcher	2 (4.9)	3 (7.5)	0	0
HCA (Health Care Assistant)	2 (4.9)	1 (2.5)	1(9)	0
GSA/WS/Workman	9 (22.0)	5 (12.5)	2(18)	1(12)
Administration/Clerk	8 (19.5)	8 (20.0)	4(37)	2(26)
Medical student	2 (4.9)	5 (12.5)	1(9)	1(12)
Retired	0 (0)	1 (2.5)		
Chaplin	0 (0)	1 (2.5)		
				
Dependent of HA/CU staff	5 (6.2)	1 (1.2)		
Non-health professional	2 (4.9)	1 (2.5)		
White collar	1 (2.4)	0(0)		
Student	2 (4.9)	0(0)		

**Baseline**				

	Mean (SD)	Mean (SD)	Mean (SD)	Mean (SD)

Age	36.0 (10.9)	35.4 (8.5)	37.1(8.8)	31.8(5.8)
Days of cough onset for this episode	3.7 (2.6)	4.3 (6.5)	2.9(1.7)	1.6(0.7)
Height (cm)	159 (6.9)	158.9 (9.0)	159.7(8.0)	162.3(3.3)
Weight (kg)	58.7 (11.8)	56.1 (8.7)	60.8(11.9)	56.9(7.1)
SBP (mmHg)	125.5 (16.3)	121.3 (16.8)	131.4(12.0)	123.9(11.4)
DBP (mmHg)	73.6 (12.1)	69.8 (9.3)	79.3(11.0)	72.5(7.5)
Pulse/min	79.5 (9.6)	77.2 (11.7)	84.4(9.0)	73.8(8.3)
Temperature (°C)	36.5 (0.4)	36.5 (0.4)	36.5(0.5)	36.6(0.3)

There were 19 subjects subsequently withdrawn from the study (characteristics shown in table 2) and the reasons were: worsening symptoms (57.9%), over-the-counter cough medicine used (21.2%), cough improved and stopped (10.4%), conditions evolved and antibiotics required (5.3%) and difficulties in taking the tablets (5.3%). Nevertheless, none of the subject had reported any adverse effect after taking TCM cough tablet.

The subjects had on average 4-days history of cough at presentation. Table 3 shows the baseline symptom severity and quality of life in physical, psychological and social domains measured by LCQ. No difference was observed in the symptoms and LCQ scores between the two groups at the start of the study. Based on clinical assessment, Fexofenadine (Telfast) was prescribed to 67 subjects (82.7%) for rhinitis, Paracetamol (Panadol) to 44 subjects (54.3%) for fever and myalgia, Benzydamine HCL (Difflam) to 15 subjects (18.5%) or Dequalinium (Dequadin) to 18 subjects (22.2%) with sore throat, Mefenamic acid (Ponstan) to 18 subjects (22.2%) with more severe myalgia, Ascorbic acid (Vitamin C) to 28 subjects (24.6%), Chlorpheniramine Maleate (Piriton) to 3 subjects (3.7%) with rhinitis, Promethazine HCL (Phenergan) to 2 subjects (2.5%) with worse nocturnal nasal symptom and Triacinolone acetonide (Kenalog in orobase) to 1 subject (1.2%) with aphthous ulcer.

**Table 3:** Comparison of baseline parameters in two study groups

	**Study Group**		
		
	TCM group N = 41 Mean (sd)	Placebo group N = 40 Mean (sd)	P-value
**Symptoms (score range 0: none – 4: very severe)**			
Day cough	1.6 (0.8)	1.5 (0.4)	0.500
Night cough	1.7 (1.1)	1.6 (1.0)	0.635
Sputum	1.5 (1.2)	1.2 (1.1)	0.228
Nasal congestion	1.6 (1.2)	1.2 (1.1)	0.895
Running nose	1.7 (1.2)	1.8 (1.4)	0.679
Sneezing	1.1 (0.9)	1.3 (1.3)	0.541
Hoarseness	1.7 (1.0)	1.6 (1.2)	0.536
Sore throat	2 (1.1)	2 (1.2)	0.842
Muscle pain	1.4 (1.2)	1.0 (1.1)	0.064
Chest pain	0.6 (0.9)	0.5 (0.8)	0.752
Headache	1.5 (1.3)	1.2 (1.4)	0.232
Total score *(sum of above 11 symptoms: 0–44)*	16.6 (6.7)	15.3 (7.0)	0.383

**QoLs (score range)**			
Physical domain (8–56)	4.7 (0.9)	4.5 (0.9)	0.278
Psychological domain (7–49)	4.9 (1.2)	4.9 (1.1)	0.955
Social domain (4–28)	4.8 (1.2)	4.7 (1.2)	0.636
Total score *(sum of above 3 domains: 3–21)*	14.4 (3.0)	14.0 (2.9)	0.616

Tables 4 and 5 show the changes of the symptoms and LCQ at Day 4 and 7. A significant improvement in symptoms and LCQ scores were observed in both the treatment and control groups during the study period, but no difference was seen between the two groups except in coughing bouts when significantly more improvement were reported in the placebo group (p = 0.027). Women taking TCM had significantly fewer problems with sputum production (p = 0.03) and older subjects (>35 years of age) reported a significant improvement in hoarseness (p = 0.05) when compared to those using placebo. (Tables 6 and 7 respectively)

**Table 4:** Change of Symptoms after taking either the TCM preparation or placebo in the studied patients

	**Study Group**									
	TCM group (N = 41)			Placebo group (N = 40)						
					
**Symptom**	***Difference between D4 and D1***	***Difference between D7 and D4***	***Difference between D7 and D1***	***Difference between D4 and D1***	***Difference between D7 and D4***	***Difference between D7 and D1***	P value^1 ^(D4–D1)	P value^1 ^(D7–D4)	P value^1 ^(D4–D1)	P value^2^*(trend difference)*
Day Cough	-0.17	-0.49	-0.66	-0.05	-0.70	-0.75	0.665	0.279	0.734	0.559
Night Cough	-0.34	-0.39	-0.73	-0.05	-0.78	-0.83	0.384	0.102	0.796	0.234
Sputum	-0.10	-0.41	-0.51	0.13	-0.20	-0.08	0.385	0.374	0.142	0.258
Nasal Congestion	-0.71	-0.27	-0.98	-0.80	-0.40	-1.20	0.749	0.541	0.502	0.763
Running Nose	-0.73	-0.24	-0.98	-0.73	-0.35	-1.08	0.982	0.664	0.730	0.911
Sneezing	-0.63	-0.10	-0.73	-0.63	-0.40	-1.02	0.973	0.178	0.273	0.399
Hoarseness	-0.76	-0.27	-1.02	-0.53	-0.55	-1.08	0.433	0.233	0.862	0.549
*Sore Throat*	-0.73	-0.49	-1.22	-0.63	-0.78	-1.40	0.738	0.286	0.523	0.605
Muscle Pain	-0.90	-0.07	-0.98	-0.70	0.00	-0.70	0.425	0.640	0.286	0.564
Chest Pain	-0.24	-0.15	-0.39	-0.40	-0.08	-0.48	0.461	0.444	0.662	0.672
Headache	-0.93	-0.20	-1.12	-0.85	-0.05	-0.90	0.783	0.503	0.468	0.718
Total Symptom Score	-6.24	-3.07	-0.92	-5.23	-4.28	-9.50	0.590	0.355	0.924	0.633

^1^*by independent student t-test; *^2^by repeated measure ANOVA;* P < 0.05

**Table 5:** Change of LCQ scoresafter taking either the TCM preparation or placebo in the studied patients

	**Study Group**		
	TCM group (N = 41)	Placebo group (N = 40)	
		
**Symptom**	***Difference between D7 and D1***	***Difference between D7 and D1***	P value^1 ^(D7–D1)
Q1 chest/stomach pain	0.41	0.65	0.489
Q2 sputum	0.24	0.70	0.260
Q3 tired	0.95	1.60	0.169
Q4 felt in control	1.05	1.10	0.908
Q5 felt embarrassed	0.80	0.53	0.488
Q6 felt anxious	0.59	0.75	0.626
Q7 interfered with daily activities	0.73	0.68	0.899
Q8 interfered with life enjoyment	0.88	0.95	0.848
Q9 paints/fumes	0.00	0.68	0.067
Q10 sleep	0.76	1.35	0.169
Q11 coughing bouts	0.32	1.18	0.027*
Q12 felt frustrated	0.54	0.63	0.808
Q13 felt fed up	0.78	0.90	0.777
Q14 hoarse voice	1.32	1.25	0.863
Q15 energy	0.95	0.45	0.066
Q16 worry	0.73	0.88	0.642
Q17 concern with other people	0.88	0.60	0.404
Q18 conversation	0.46	0.90	0.267
Q19 annoyed friends	0.80	0.88	0.853

**Physical Domain**	0.62	0.98	0.161

**Psychological Domain**	0.77	0.77	0.996

**Social Domain**	0.72	0.85	0.683

**Total Score**	2.11	2.60	0.543

^1^by independent student t-test; * P < 0.05

**Table 6:** Differences in symptoms between the TCM and placebo groups in female subjects

	**Study Group**		
	TCM Group	Placebo Group	
		
**Parameters**	*Median (range)*	*Median (range)*	*P Value*
**Symptoms**			
Day Cough	-1 (-3 – 4)	-1 (-3 – 3)	0.958
Nigh Cough	-1 (-3 – 4)	-1 (-3 – 3)	0.910
Sputum	-1 (-4 – 4)	0 (-2 –4)	0.029*
Nasal Congestion	-1 (-3 – 2)	-1 (-4 – 3)	0.749
Running Nose	-1 (-3 – 4)	-1 (-3 – 3)	0.944
Sneezing	-1 (-3 – 3)	-1 (-3 – 1)	0.629
Hoarseness	-1 (-3 – 3)	-1 (-3 – 2)	0.594
Sore Throat	-1 (-3 – 2)	-1 (-4 – 1)	0.517
Muscle Pain	-1 (-4 – 1)	0 (-3 – 1)	0.374
Chest Pain	0 (-4 – 1)	0 (-3 – 0)	0.937
Headache	-1 (-4 – 1)	0 (-4 – 2)	0.510
**Total Score**	-11 (-26 – 16)	-10 (-27 – 15)	0.722

= visit 3 – visit 1

**Table 7:** Differences in symptoms between the TCM and placebo groups in subjects >35

	**Study Group**		
	TCM Group	Placebo Group	
		
**Parameters**	*Median (range)*	*Median (range)*	*P Value*
**Symptoms**			
Day Cough	0 (-3 – 1)	0 (-2 – 3)	0.163
Nigh Cough	0 (-3 – 2)	0 (-3 – 4)	0.885
Sputum	0 (-3 – 1)	0 (-2 – 3)	0.261
Nasal Congestion	-1 (-3 – 0)	0 (-3 – 2)	0.250
Running Nose	-1 (-2 – 1)	0 (-3 – 2)	0.281
Sneezing	-1 (-3 – 1)	0 (-3 – 1)	0.064
Hoarseness	-1 (-4 – 3)	0 (-2 – 1)	0.048*
Sore Throat	-1 (-3 – 3)	-1 (-3 – 4)	0.965
Muscle Pain	-1 (-4 – 0)	0 (-3 – 1)	0.159
Chest Pain	0 (-3 – 0)	0 (-3 – 1)	0.859
Headache	-1 (-3 – 0)	0 (-4 – 2)	0.235
**Total Score**	-8.5 (-24 – 9)	-4 (-21 – 19)	0.143

= visit 3 – visit 1

## Discussion

The present study aimed to look for an effective, safe and affordable alternative treatment of acute cough resulted from uncomplicated URTI. The results of this study confirmed that URTI was a usually self-limiting disease with its symptoms improved in the first week of presentation. However, the herbal combination used in this study showed it did not improve symptoms when compared to the placebo. This formulary was well tolerated with no adverse effects reported. This finding had significant clinical implications for the Chinese population because this formulary shared many components in commercially available ready-made TCM preparations in the market (for example, Pei Pa Kao). Other components of these preparations might have significant roles to play such as the soothing effect of a syrup preparation. In addition, this study also highlighted some difficulties when conducting a TCM research in a western clinical setting as discussed below.

Firstly, this study covered mainly young working adults, who had expected western treatment. There was an added possibility that, in a fast paced society such as Hong Kong, immediate relief of symptoms might outweigh the other advantages of TCM. This might account for high drop-out rates and the demand for concomitant use of other relieving medication such as antihistamine, NSAIDs and vitamin C, which could potentially have confounding effects on relieving cough symptoms of the TCM preparation. For example, cough induced by post-nasal drip could be reduced by the anti-histamines. However, clinicians usually found that, in reality, patients had expected the other symptoms to be controlled at first presentation and this reflected how the studied drug would be used in a normal clinical setting. Secondly, one might argue that it would be better to compare the TCM in study with a commonly used cough medicine, for example, dextromethorphan. However, it would be very difficult to blind the subjects as they were of very different preparation with different odour and taste (the TCM had its own distinct favour), and when the effectiveness of currently available cough medicine were in question. Thirdly, the symptoms of URTI were usually very subtle and hence a very sensitive tool might be required to measure the changes in such a short period of time. LCQ was originally designed to measure changes in chronic cough and no tools tracking changes in acute cough was available.

Available research and evidence of using TCM in treating cough was limited and only two studies were found in literature search using Medline: one on tumeric oil [19] and the other on Feiyan Chuansou Oral Liquid (FCOL) [20]. It was found that turmeric volatile oil was significantly active in removing sputum, relieving cough and preventing asthma. FCOL were significantly better in its antitussive, expectorant, anti-asthmatic effect and resolution of dry and moist rale, and wheezing in treatment group than those in the control group. On the other hand, a recent study found the popular Echinacea to be ineffective in treating URTI in children age from 2 to 11 years old in the USA [21]. In all these studies, a standard herbal formula was used to irrespective of "TCM differentiation". "TCM differentiation" was the fundamental to TCM care and treatment whereby TCM practitioners would choose different formulas for different types of cough based on TCM diagnoses made for individual patients. Many TCM research including this one used fixed formula and did not deal with the rationale of such choice and hence the appropriation of the formulation is questionable. On the other hand, if we were to assess the efficacy of Chinese medicine without standardisation, this lack of standardisation would introduce many confounding variables and make comparison impossible.

In Hong Kong, polypharmacy (a URTI patient received an average of 1.3 cough medicine) and using dangerous drugs such as theophylline or steroid at the risk of developing drugs interactions, side effects or complications, was common in the management of URTI [7]. The high prevalence and morbidity of this illness as well as its economic and social implications warrant further search for an effective treatment and measuring tools in this area.

## Conclusion

In order to coincide with government's effort in reducing use of antibiotics to treat URTI, patients were encouraged not to seek medical help at the first instance but try to self-medicate for their symptoms. However, evidence on the effectiveness of different types of anti-tussive was inconclusive. At best, only a limited number of anti-tussive such as dextromethorphan and guaifenesin may be helpful in relieving this symptom. Since Chinese herbal medicine was widely used and accepted in Hong Kong, it might provide a good base to look into its role in relieving acute cough symptom as some TCM ingredients had already been shown to be effective. The studied combination in the tablet preparation did not show any overall self-reported improvement in terms of acute symptoms or quality of life. Other factors such as syrup preparation or placebo effects might have contributed to the popularity of herbal cough medicine available to the general public. Further search for a safe and effective TCM was warranted.

## Abbreviations

URTI – Upper Respiratory Tract Infections

TCM – Traditional Chinese Medicine

LCQ – Leicester cough questionnaire

## Competing interests

The author(s) declare that they have no competing interests.

## Authors' contributions

WCWW and ELYW designed, supervised and wrote up the report. PCL designed and arranged the TCM for the study. AL advised on the design of the protocol and supervises the study. ATL, KTL and CYML conducted the study in the clinic. JLT advised on the biostatistics.

## Acknowledgements

The authors would like to acknowledge Dr Surinder Birring, Department of Respiratory Medicine, Glenfield Hospital, Leicester LE3 9QP, United Kingdom who has kindly allowed us to use the LCQ in this study.
